# Person‐centred HIV care and prevention for youth in rural South Africa: preliminary implementation findings from *Thetha Nami ngithethe nawe* stepped‐wedge trial of peer‐navigator mobilization into mobile sexual health services

**DOI:** 10.1002/jia2.70032

**Published:** 2025-10-08

**Authors:** Jacob Busang, Nqobile Ngoma, Thembelihle Zuma, Carina Herbst, Nonhlanhla Okesola, Natsayi Chimbindi, Jaco Dreyer, Theresa Smit, Kristien Bird, Lucky Mtolo, Osee Behuhuma, Willem Hanekom, Kobus Herbst, Limakatso Lebina, Janet Seeley, Andrew Copas, Kathy Baisley, Maryam Shahmanesh

**Affiliations:** ^1^ Africa Health Research Institute Mtubatuba South Africa; ^2^ Institute for Global Health University College London London UK; ^3^ School of Medicine Faculty of Health Sciences University of Pretoria Pretoria South Africa; ^4^ University of KwaZulu‐Natal Durban South Africa; ^5^ DSI‐SAMRC South African Population Research Infrastructure Network (SAPRIN) Durban South Africa; ^6^ University of the Witwatersrand Johannesburg South Africa; ^7^ London School of Hygiene & Tropical Medicine London UK

**Keywords:** person‐centred, HIV prevention, pre‐exposure prophylaxis, adolescents and young adults, sexual and reproductive health, South Africa

## Abstract

**Introduction:**

Despite the efficacy of antiretroviral therapy (ART)‐based prevention, population‐level impact remains limited because those at high risk of HIV acquisition are not reached by conventional services. We investigated whether youth‐centred and tailored HIV prevention, delivered by community‐based peer navigators alongside sexual and reproductive health (SRH) services, can mobilize demand for HIV pre‐exposure prophylaxis (PrEP) and ART among adolescents and young adults (AYA) in KwaZulu‐Natal, South Africa.

**Methods:**

*Thetha Nami ngithethe nawe* is a cluster‐randomized stepped‐wedge trial (SWT) in 40 clusters within a rural health and demographic surveillance site. Clusters were randomized to receive the intervention in period 1 (early) or period 2 (delayed). Trained area‐based peer navigators conducted needs assessments with youth aged 15–30 years to tailor health promotion, psychosocial support and referrals into nurse‐led mobile SRH clinics that also provided HIV testing, and status‐neutral ART and oral PrEP. Standard of care was PrEP delivered through primary health clinics. We report SRH service uptake from the 20 intervention clusters during the first period of the SWT (NCT05405582).

**Results:**

Between June 2022 and September 2023, peer‐navigators reached 9742 (74.9%) of the 13,000 youth in the target population, 46.8% males. Among 9576 individuals with needs assessment, peer‐navigators identified 141 (1.5%) with social needs, and 4138 (43.5%) had medium to high health needs. These individuals were referred to mobile clinics, with 2269 (54.8%) attending, including 959 (42.3%) males. HIV testing uptake was high (92.7%; 2103/2269), with 10.1% (212/2103) testing positive for HIV, 62 (29.2%) of whom started ART for the first time. The prevalence of HIV was higher among females compared to males (15.1% vs. 3.3%; *p* < 0.001). Among clinic attendees, 96.8% were screened for PrEP eligibility, with 38.5% deemed eligible and offered PrEP. Of the 1433 (63.2%) individuals tested for sexually transmitted infections (STIs), 418 (29.2%) tested positive, with females having higher STI prevalence (37.2% vs. 17.9%; *p* < 0.001). Of these, 385 (92.1%) received STI treatment. Among 1310 females, 769 (58.7%) reported not using any contraception at their initial visit, and 275/769 (35.8%) started contraception during the trial.

**Conclusions:**

Community‐based and person‐centred approaches delivered through trained peer‐navigators can link AYA with SRH and HIV prevention/care needs with mobile SRH services.

## 1 INTRODUCTION

South Africa has the largest HIV epidemic globally, with an estimated 7.8 million people living with HIV (PLHIV) in 2022 [1]. Despite expanded HIV programmes offering effective and free biomedical interventions, such as HIV testing, universal test and treat (UTT), and oral pre‐exposure prophylaxis (PrEP), HIV incidence remains high among young people, particularly adolescent girls and young women (AGYW) aged 20–24 in rural KwaZulu‐Natal (KZN) [2, 3].

Antiretroviral therapy (ART)‐based HIV prevention strategies have demonstrated efficacy at the individual level but have not yet achieved population‐level impact in southern Africa [4], particularly among adolescents and young adults (AYA). Barriers to access include gender and sexuality norms, HIV stigma, and the time and cost of facility‐based care [5, 6, 7]. Community‐based and peer‐led interventions can help overcome these barriers by generating demand and improving delivery of sexual and reproductive health (SRH) services, including PrEP [8, 9, 10, 11]. Such approaches often adopt person‐centred care (PCC), which is associated with improved PrEP uptake [12] and treatment adherence [13, 14]. PCC is endorsed by the Joint United Nations Programme on HIV/AIDS (UNAIDS) through its 2025 targets, emphasizing context‐specific, comprehensive services and removal of societal and legal barriers to access [15].

Peer‐led HIV prevention is well‐supported among key populations such as sex workers, men who have sex with men and people who inject drugs, with one review reporting a 36% reduction in HIV incidence [10]. However, evidence among AYA, particularly in sub‐Saharan Africa, remains limited. Of 54 peer‐based HIV prevention interventions reviewed, only 12 targeted youth, mostly using school‐based education or participatory learning to influence knowledge and behaviour, without evaluating biomedical outcomes [16]. Moreover, few studies have assessed the population‐level impact of peer navigator models delivering person‐centred SRH services to youth in high‐prevalence, rural settings [10].

Between 2019 and 2021, we co‐created and adapted a peer navigator‐led intervention with AYA to support engagement with person‐centred HIV care and prevention, including oral PrEP in rural KZN [5, 17, 18, 19, 20]. The resulting intervention, *Thetha Nami ngithethe nawe* (loosely translated as “Let's Talk”), is currently being evaluated in a stepped‐wedge cluster randomized trial (SW‐CRT) for its effectiveness, implementation and cost‐effectiveness in increasing uptake of oral PrEP and reducing sexually transmissible HIV among 15‐ to 30‐year‐olds [21].

This paper reports early descriptive implementation findings from the first trial period in 20 intervention clusters. The intervention aligns with PCC principles by addressing unmet health and social needs through tailored HIV and SRH services delivered via community‐based peer support and mobile clinics. Here, we describe patterns of service uptake, focusing on the reach of HIV testing, sexually transmitted infection (STI) screening and treatment, and PrEP offer and initiation among AYA in these early clusters.

## 2 METHODS

### 2.1 Overview of *Thetha Nami Ngithethe Nawe* (Let's Talk) trial

*Thetha Nami ngithethe nawe (Thetha Nami* for short) has been described in detail elsewhere [21]. Briefly, it is an SW‐CRT evaluating the implementation and effect of a peer navigator–led, biosocial HIV prevention intervention targeting young people aged 15–30 in rural KZN, South Africa (trial registration NCT05405582). Forty clusters (administrative areas) were randomized in a public ceremony to receive the intervention either in the first period (“early”) or in the second period (“delayed”), with rollout staggered over two 24‐month periods. Randomization was restricted to ensure balance by population size of 15‐to 30‐year‐olds, geographic region of the study area (north vs. south) and proximity to a major road.

Each cluster had a pair of resident peer navigators delivering the intervention. The first 20 clusters began implementation in June 2022 and will continue receiving the intervention until the planned study end date in January 2026. The second step (remaining clusters) began in September 2023 and will also continue until the study concludes in January 2026. During the first period of the stepped‐wedge rollout, the delayed clusters did not receive any study‐related outreach or intervention activities. These clusters continued to access routine services through public health facilities, and no peer navigator engagement occurred until their scheduled intervention rollout.

### 2.2 Setting

*Thetha Nami* is embedded in the Africa Health Research Institute's (AHRI) health and demographic surveillance system (HDSS) in uMkhanyakude district, rural KZN [22]. The area has approximately 160,000 residents, including around 26,000 aged 15–30 years with high youth unemployment (> 85%) and HIV burden. Our previous studies have shown that there is a high unmet sexual health need among young people, including low engagement with existing HIV prevention and care services in this setting [7, 19, 20, 23, 24, 25]. The area has 11 primary healthcare (PHC) clinics, which have started to provide free oral PrEP since 2021. All people residing in AHRI HDSS have a unique identifier enabling us to link young people who engage with peer navigators to the study clinics.

### 2.3 Study population

All 15‐ to 30‐year‐olds residing in 40 clusters are eligible to receive the intervention. Based on our pilot work using a screening tool aligned with South African national guidelines, an estimated 16% of this population is at risk of HIV acquisition and would benefit from PrEP.

### 2.4 Study interventions

Clusters were randomly assigned to early or delayed rollout of the *Thetha Nami* intervention. Delayed clusters received standard of care (SOC) during the first period of the trial.

### 2.5 SOC clusters

In SOC clusters, participants had access to free nurse‐led HIV prevention and treatment services at PHCs within the surveillance area, including HIV counselling and point‐of‐care testing, with immediate initiation of ART for individuals testing positive or PrEP for eligible HIV‐negative individuals following South African National PrEP guidelines. According to these guidelines, individuals initiated on PrEP are scheduled for follow‐up visits at 1 month and every 3 months thereafter for repeat HIV testing, counselling, adherence support and prescription refills. Similarly, individuals initiated on ART attend follow‐up visits every 3 months for prescription refills and adherence counselling, with viral load monitoring conducted at 6‐ and 12‐months post‐initiation and annually thereafter if viral suppression is maintained. In addition, SRH services, such as family planning support and syndromic management of STIs, were also provided in line with the South African National Department of Health guidelines.

### 2.6 Thetha Nami ngithethe nawe intervention clusters

This is a peer‐led, person‐centred intervention offering tailored psychosocial support and mobilizes youth into community‐based SRH services. It also facilitates differentiated HIV prevention and care, including PrEP and UTT. Peer navigators are 18‐ to 30‐year‐olds residing in the clusters in which they work and who have completed high school. They are selected through a process of referral by traditional and municipal leaderships and assessments. They undergo 6–8 weeks of formal classroom training in a range of core competencies: namely certification for HIV counselling and testing; SRH, HIV treatment and PrEP health promotion; child protection, confidentiality and good clinical practice; youth development; community engagement and strategies for safely entering homesteads; and use of an electronic clinical management tool in REDCap. Training incorporates conducting needs assessments, role play activities, facilitating safe spaces with young people, and weekly debriefings with a review panel including nurse and social worker support. Approximately 90 peer navigators work part‐time within the intervention clusters, supported by supervisors (experienced peer navigators), a review panel and a mobile sexual health clinic that visits areas designated by the peer navigators monthly.

Peer navigators conduct community mobilization through events and safe spaces, engage youth in broad health promotion conversations, and perform needs assessments (health, social, legal or education) and triage the urgency of the need (low to high):

- Low need: provide health promotion and a toll‐free clinical hotline to link with the clinic or peer navigator should a need arise prior to the scheduled 3‐month follow‐up.
- Medium need: referral to clinical or social services and a scheduled follow‐up for within a week.
- High need: immediate escalation to the social worker or nurse with a follow‐up within 3 days.

Based on these holistic assessments, they agree on an action plan including health promotion and counselling, referrals and follow‐up scheduling. All interactions and plans are recorded electronically, which enables real‐time integration with clinical and social services.

The mobile clinics are nurse‐led, HIV‐status neutral, gender‐neutral and provide adolescent‐ and youth‐friendly HIV testing, prevention and care, integrated with SRH services, including point‐of‐care HIV testing with confirmatory testing and ART initiation as needed. PrEP eligibility is assessed following national guidelines. STI testing for syphilis, hepatitis B, gonorrhoea and chlamydia is offered with treatment and partner notification. PrEP or ART initiators receive a monthly supply and follow‐up care, including a 7‐day post‐initiation phone call and subsequent clinic visits at 1 month and then every 3 months. Full intervention details and protocols have been published elsewhere [21].

### 2.7 Data collection

Between 6 June 2022 and 18 September 2023, process data were collected on peer navigator outreach, needs assessments, referrals, clinic attendance, and uptake of HIV and SRH services. The outcomes reported in this paper reflect data from the 20 early intervention rollout clusters at the end of the first period of the stepped‐wedge trial. Based on population enumeration data from the AHRI HDSS, the 20 clusters comprise approximately 13,000 AYA (aged 15–30 years), representing half of the total study population enrolled in the trial. Key measures include HIV testing and the offer of PrEP and ART, seroconversion rates, testing for common and curable STIs, and contraception uptake among females.

### 2.8 Statistical analysis

We conducted descriptive analyses to evaluate the uptake and outcomes of HIV prevention and SRH services by sex and age among early intervention clusters. Categorical variables are presented as frequencies and percentages; continuous variables as medians and interquartile range. Analyses accounted for clustering by peer navigator catchment (the unit of randomization and intervention delivery) using Stata's *svy* commands with Taylor‐linearized variance estimation for standard errors. Sex‐specific differences in proportions were assessed using design‐based Pearson's chi‐square tests with Rao–Scott corrections. For continuous variables, mean differences were tested using linearized standard errors under the *svy* framework.

Key outcomes analysed included:

1. HIV testing, seroconversion, PrEP uptake and ART initiation.
2. STI testing, prevalence of common and curable STIs (chlamydia, gonorrhoea and trichomonas) and treatment.
3. Contraceptive use and receipt during the trial. New contraceptive users were defined as female participants who received a contraceptive method during their visit to a study clinic and had reported not using any contraception at the time of their initial clinic visit.
4. Pregnancy during the study.

All analyses were stratified by sex and age to assess sex‐specific trends. Data were analysed using Stata 18.

### 2.9 Ethical considerations

The study followed ethical guidelines per the Declaration of Helsinki. Ethical approvals were obtained from the University of KwaZulu‐Natal Biomedical Research Ethics Committee (BREC/00003735/2021) and UCL Research Ethics Committee (5672/006). The study team, including the implementing staff, received ethics training covering confidentiality, voluntary participation and good clinical practice. Each participant was assigned a unique, non‐identifying participant identification number to ensure confidentiality. Participants were provided with detailed information about the study and were given the opportunity to ask questions for clarification before providing consent. Participants aged 18+ provided written informed consent; those aged 15–17 provided assent with parental/guardian consent. Participants could withdraw from the study at any time without consequences. All study services adhered to national and clinical guidelines.

## 3 RESULTS

### 3.1 Peer navigators reach and need assessments

**Figure 1:**
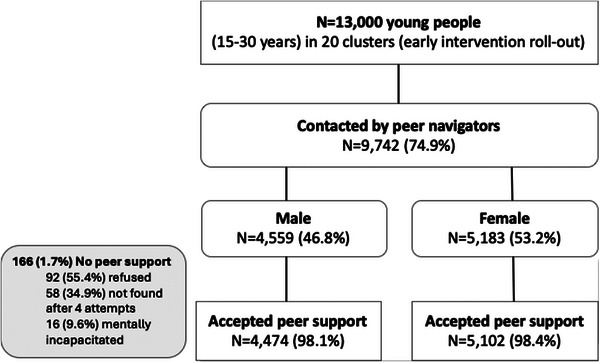
Peer navigator reach.

Peer navigators reached 9742 (74.9%) of the 13,000 young people in the target population across the 20 clusters with early intervention rollout (Figure 1). Of those reached, 46.8% were male participants. A total of 9576 (98.3%) young people accepted peer support and underwent needs assessment.

**Figure 2:**
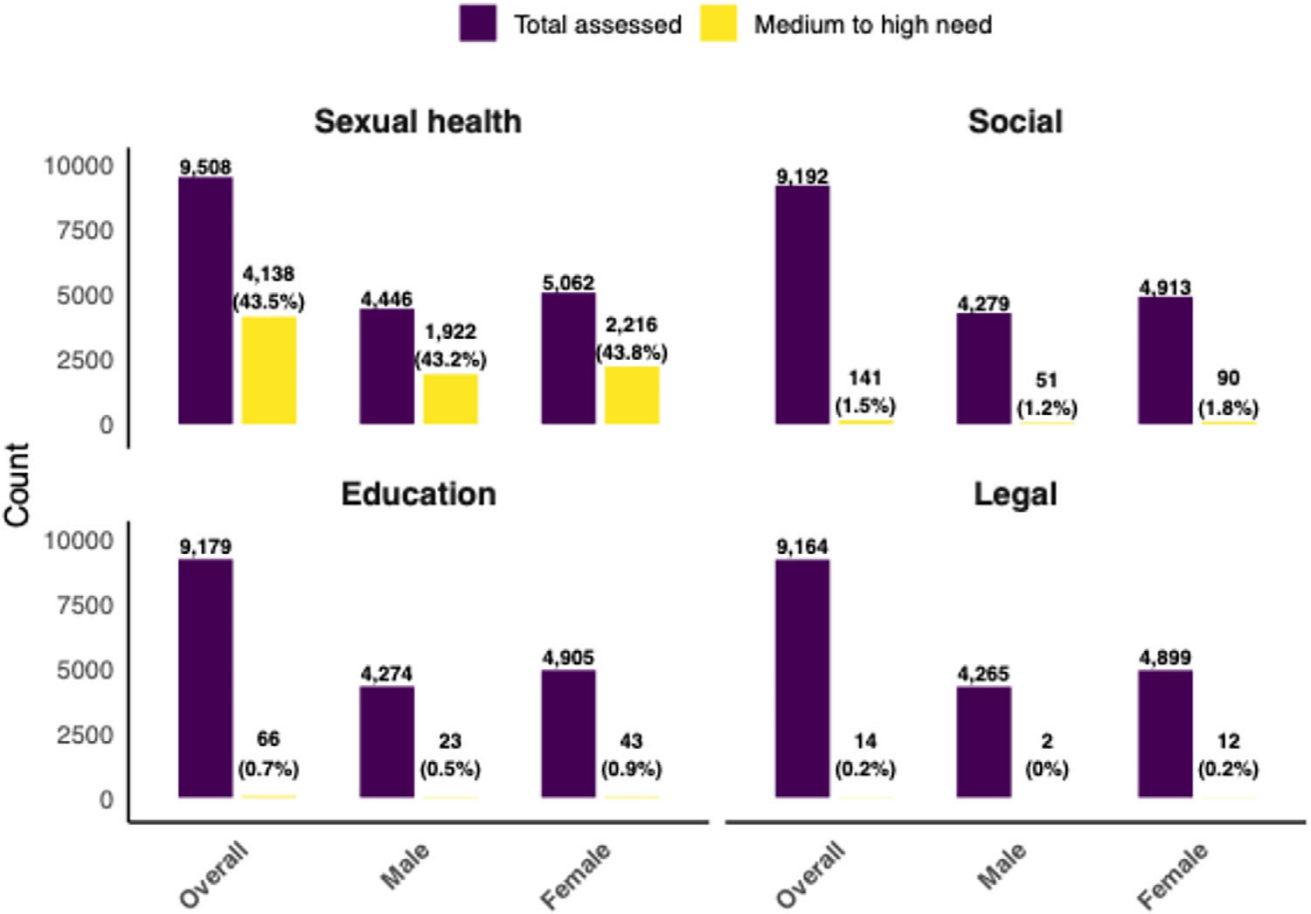
Needs assessment and urgency grading, overall and by sex. Observed differences in denominators across domains reflect occasional missing responses to specific items within the needs assessment tool.

Among the young people assessed, sexual health needs were the most frequently reported, with 9508 (99.2%) assessed, of whom 4138 (43.5%) were identified as having medium to high needs and were referred for mobile adolescent and youth‐friendly clinical services. In contrast, assessments for social support, education and legal needs revealed much lower proportions of medium or high need: 1.5% (141/9192), 0.7% (66/9179) and 0.2% (14/9164), respectively, and were referred for social, educational or legal services as needed. The patterns of needs were similar across sexes. Among females, 43.7% (2216/5062) had medium or high sexual health needs compared to 43.2% (1922/4446) among males (Figure 2).

### 3.2 Participants who attended mobile adolescent and youth‐friendly clinical services

Among the 4138 individuals identified with medium or high sexual health needs and referred for clinical services, 2269 (54.8%) successfully linked to care. Linkage rates were higher among females, with 59.1% (1310/2216) successfully linking to care, compared to 49.9% (959/1922) among males. There were more females than males across all age groups. About one in five participants were residing in urban or peri‐urban areas (Table 1).

**Table 1:** Demographic characteristics of the participants who attended mobile adolescent and youth‐friendly clinical services

	Total		Male		Female		
	*n* = 2269		*n* = 959		*n* = 1310		*p*‐value
**Age at enrolment (years)**							0.010
Median [IQR]	21.7	[18, 26]	21.0	[18, 25]	22.1	[19, 26]	
**Age group**							0.06
15–19	860	37.9%	394	41.1%	466	35.6%	
20–24	783	34.5%	328	34.2%	455	34.7%	
25–30	626	27.6%	237	24.7%	389	29.7%	
**Area of residence**							0.05
Rural	1789	78.9%	775	80.9%	1014	77.5%	
Urban/peri‐urban	477	21.1%	183	19.1%	294	22.5%	

Abbreviation: IQR, interquartile range.

### 3.3 Uptake of HIV testing and assessment for HIV prevention and care

Table 2 presents uptake of HIV testing by sex. A total of 2103 (92.7%) of clinic attendees underwent HIV testing, with a similar uptake rate of HIV testing between males (93.4%) and females (92.1%). Of those tested, 212 (10.1%) were living with HIV; this was higher in females (15.1%) than males (3.3%), *p* < 0.001. Among those diagnosed as living with HIV, 62 (29.2%) were newly diagnosed and started on ART, with more males (46.7%) than females (26.4%) newly diagnosed (*p* = 0.05). Repeat HIV testing was conducted in 28.2% (*n* = 594) of participants, with a higher proportion among females (30.4%) compared to males (25.3%; *p* = 0.02). Seroconversion occurred in 1.0% (*n* = 6) of participants who retested, five of whom were female. The overall HIV incidence rate was 2.53 per 100 person‐years (95% CI: 1.13–5.62). Among the 2269 clinic attendees, 2196 (96.8%) were screened for PrEP eligibility, and 845 (38.5%) were deemed eligible and offered PrEP. More males (46.1%) than females (32.9%) were offered PrEP. Uptake of PrEP was 85.3% among 845 who were offered PrEP and did not differ by sex (86.5% among males vs. 83.7% among females, *p*  =  0.42).

**Table 2:** Uptake of HIV testing and assessment for HIV prevention and care among adolescents and young adults in rural South Africa, June 2022–September 2023

	Total		Male		Female		
	*n* = 2269		*n* = 959		*n* = 1310		*p*‐value
**HIV testing**							
DBS collected for HIV testing							0.27
No	166	7.3%	63	6.6%	103	7.9%	
Yes	2103	92.7%	896	93.4%	1207	92.1%	
HIV status							< 0.001
Negative	1891	89.9%	866	96.7%	1025	84.9%	
Positive	212	10.1%	30	3.3%	182	15.1%	
ART status							0.05
On ART	150	70.8%	16	53.3%	134	73.6%	
Started/initiated ART	62	29.2%	14	46.7%	48	26.4%	
Tested at least twice							0.02
No	1509	71.8%	669	74.7%	840	69.6%	
Yes	594	28.2%	227	25.3%	367	30.4%	
Seroconverted							0.31
No	588	99.0%	226	99.6%	362	98.6%	
Yes	6	1.0%	1	0.4%	5	1.4%	
**PrEP eligibility assessment**							
Assessed for PrEP eligibility							0.45
No	73	3.2%	26	2.7%	47	3.6%	
Yes	2196	96.8%	933	97.3%	1263	96.4%	
Considered suitable for PrEP following risk assessment							< 0.001
No	1351	61.5%	503	53.9%	848	67.1%	
Yes	845	38.5%	430	46.1%	415	32.9%	
Started PrEP							0.42
No	124	14.7%	58	13.5%	66	15.9%	
Yes	721	85.3%	372	86.5%	349	84.1%	
**Common curable STI testing**							
Tested for STI							0.42
No	836	36.8%	364	38.0%	472	36.0%	
Yes	1433	63.2%	595	62.0%	838	64.0%	
Any STI^a^							< 0.001
Negative	1012	70.8%	486	82.1%	526	62.8%	
Positive	418	29.2%	106	17.9%	312	37.2%	
Number treated for STI^b^	385	92.1%	99	93.4%	286	92.0%	0.67

Abbreviations: ART, antiretroviral therapy; DBS, dried blood spot; PrEP, pre‐exposure prophylaxis; STI, sexually transmitted infection.

^a^ Chlamydia, gonorrhoea or trichomonas.

^b^ Among 33 participants who were not treated for a positive STI, 19 (57.5%) could not be traced (lost to follow up), 7 (21.2%) migrated out of the study area, 1 (3.0%) refused treatment, 6 (18.2%) other.

### 3.4 Uptake of STI testing and prevalence of common and curable STIs

A total of 1433 (63.2%) clinic attendees were tested for common and curable STIs (chlamydia, gonorrhoea and trichomonas) with similar proportions for males (62.0%) and females (64.0%) (Table 2). Overall, 418 (29.2%) tested positive for any STI, with a higher prevalence among females (37.2%, 95% CI: 34.4%–40.1%) than males (17.9%, 95% CI: 15.4%–20.8%) (Table S1). Among those who tested positive, 385 (92.1%) received STI treatment. Treatment uptake did not differ significantly by sex (*p* = 0.67).

**Figure 3:**
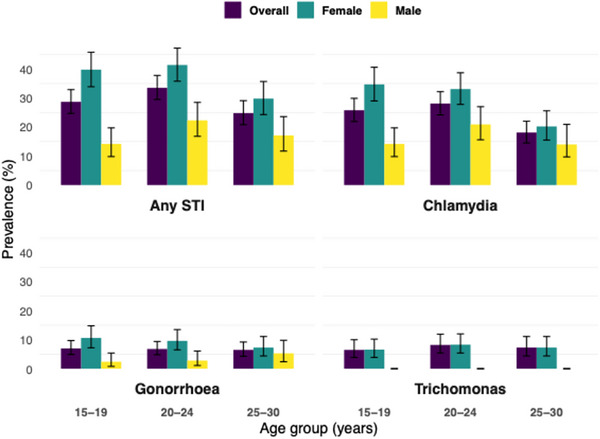
Prevalence of sexually transmitted infections (STI) by sex and age group.

Across all age groups, the prevalence of any STI was consistently higher among females compared to males, and it was highest in females aged 15–24 and men aged 20–24 (Figure 3 and Table S2). For instance, in the 15–19 age group, 39.8% (95% CI: 35.3%–44.8%) of females tested positive for any STI compared to 14.2% (95% CI: 10.1%–19.6%) of males. Similarly, among females aged 20–24 years, 41.4% (95% CI: 37.2%–45.7%) tested positive for any STI, compared to 22.3% (95% CI: 16.0%–30.1%) of males.

Chlamydia was the most prevalent STI, with an overall prevalence of 25.8% (95% CI: 21.8%–30.2%) in the 15–19 age group, declining to 18.1% (95% CI: 14.6%–22.1%) in the 25–30 age group. Among females, the highest prevalence of chlamydia was observed in the 15–19 age group, at 34.7% (95% CI: 30.4%–39.2%). Among males, the highest prevalence of chlamydia was in the 20–24 age group, at 20.9% (95% CI: 15.0%–28.2%). The prevalence of gonorrhoea was markedly lower, with overall rates of 7.0% (95% CI: 4.8%–10.1%) in the 15–19 age group and 6.5% (95% CI: 4.2%–9.9%) in the 25–30 age group. Trichomonas acquisitions were rare, with the highest prevalence observed among females aged 20–24 years at 8.3% (95% CI: 5.5%–12.3%).

### 3.5 Contraception use and pregnancy among female participants

Contraception use was reported by 65.6% (859/1310) of female participants. Among various age groups, contraception use was most prevalent in the 25–30 age group, where 79.7% (310/389) of participants reported current contraception use. Overall, 65.0% (558/1310) of contraception users received contraception through the study, with higher uptake rates among those in the 15–19 age group (70.5%, 148/210). The majority (72.8%, 406/558) of the participants who received contraception from our study clinics received injectable contraception. Among the 769 participants who were not using contraception at their initial clinic visit, 275 (35.8%) initiated a method during the study. New use was highest among participants aged 25–30 years (49.7%), followed by those aged 20–24 (41.9%) and 15–19 (25.3%) (*p* = 0.001). A total of 905 (69.1%) had a point‐of‐care test for pregnancy. Of these, 48 (5.3%) tested positive for pregnancy, with similar rates across the age groups. When including those who self‐reported current pregnancy, the number of pregnant individuals increased to 118 (9.0%), again with no substantial differences by age groups (Table 3).

**Table 3:** Contraceptive use and pregnancy among female adolescents and young adults in rural South Africa

	Overall	15–19	20–24	25–30	
	*n* = 1310	*n* = 466	*n* = 455	*n* = 389	*p*‐value
**Contraception use**					
Self‐report current contraception use at initial clinic visit					
No	769 (58.7)	364 (78.1%)	234 (51.4%)	171 (44.0%)	< 0.001
Yes	541 (41.3%)	102 (21.9%)	221 (48.6%)	218 (56.0%)	
Self‐report current contraception use at any time during the study					
No	451 (34.4%)	256 (54.9%)	116 (25.5%)	79 (20.3%)	< 0.001
Yes	859 (65.6%)	210 (45.1%)	339 (74.5%)	310 (79.7%)	
Received contraception from study clinics					
No	301/859 (35.0%)	62/210 (29.5%)	135/339 (39.8%)	104/310 (33.5%)	0.09
Yes	558/859 (65.0%)	148/210 (70.5%)	204/339 (60.2%)	206/310 (66.5%)	
Injectable contraception	406 (72.8%)	107 (72.3%)	158 (77.5%)	141 (68.5%)	0.26
Oral contraception	152 (27.2%)	41 (27.7%)	46 (22.6%)	65 (31.6%)	
Started contraception during the study					
No	494/769 (64.2%)	272/364 (74.7%)	136/234 (58.1%)	86/171 (50.3%)	0.001
Yes	275/769 (35.8%)	92/364 (25.3%)	98/234 (41.9%)	85/171 (49.7%)	
**Pregnancy**					
Point of care test for pregnancy done					
No	405 (30.9%)	126 (27.0%)	146 (32.1%)	133 (34.2%)	0.20
Yes	905 (69.1%)	340 (73.0%)	309 (67.9%)	256 (65.8%)	
Pregnancy test results					
Negative	856/904 (94.7%)	321/340 (94.4%)	290/308 (94.2%)	245/256 (95.7%)	0.73
Positive	48/904 (5.3%)	19/340 (5.6%)	18/308 (5.8%)	11/256 (4.3%)	
Self‐report currently pregnant					
No	1174/1280 (91.7%)	419/451 (92.9%)	412/449 (91.8%)	343/380 (90.3%)	0.46
Yes	106/1280 (8.3%)	32/451 (7.1%)	37/449 (8.2%)	37/380 (9.7%)	
Positive pregnancy test results or self‐reported currently pregnant					
No	1192/1310 (90.1%)	428/466 (91.9%)	413/455 (90.8%)	351/389 (90.2%)	0.72
Yes	118/1310 (9.0%)	38/466 (8.1%)	42/455 (9.2%)	38/389 (9.8%)	

## 4 DISCUSSION

Community‐based, peer‐led, PCC achieved substantial reach, engaging about 75% of the target population and ensuring equitable sexual health needs assessments across sexes. Among those who linked to care, there was a high burden of SRH and HIV care and prevention needs, highlighting the potential of peer‐led needs assessments to mobilize demand for SRH and differentiated HIV care and prevention services. However, the trial's final outcomes are needed to determine the population‐level impact of the intervention.

We found that more than half of the AYA, including men who linked to care through this person‐centred community‐based approach, were at high risk of HIV acquisition or transmission and would potentially benefit from ART‐based prevention. Our preliminary findings are consistent with a 2021 Zambian study evaluating a peer‐led, community‐based SRH intervention, which reported a significant increase in service uptake among AYA. In the intervention arm, 64% accessed SRH services compared to only 5% in the control arm [26]. Emotional dynamics, lived experiences and shared personal characteristics influence young people's decisions to engage with care, demonstrating the importance of tailored, PCC [27].

Peer navigators, embedded in their communities, significantly enhanced SRH and HIV service uptake in part by being relatable to the target population, sharing cultural, linguistic, and socio‐economic contexts and supporting their agency in navigating services [28, 29]. This model can potentially address health inequities and reproductive justice [29]. However, only just over half of those referred into services by the peer‐navigators utilized the accessible youth‐friendly SRH services, suggesting further barriers, such as internal and external stigma and societal and cultural taboos around adolescent and youth sexuality. These barriers need to be overcome to really provide equitable and timely access to sexual health and HIV prevention services to young people in these rural communities.

We observed a high prevalence of curable STIs, particularly gonorrhoea and chlamydia, with higher rates among females and those under 24. Given the SRH complications associated with untreated STIs and their frequently asymptomatic nature, this signals the urgent need for enhanced STI screening, testing, contact tracing and early treatment models. Consistent with our findings, a 2019 systematic review and meta‐analysis on the global epidemiology of STIs among PrEP users reported that nearly one‐quarter of patients at PrEP initiation had an STI (chlamydia, gonorrhoea or early syphilis). Furthermore, the study documented an STI incidence rate of 72.2 per 100 person‐years during the first 3 months of PrEP use [30].

This person‐centred and community‐based delivery approach substantially reached AGYW with unmet contraceptive needs, with a significant proportion, particularly among adolescents, initiating use through the mobile sexual health services. These findings mirror a Kenyan study that reported a 12.5% increase in contraceptive use paired with a significant decrease in pregnancy incidence associated with integrated family planning into HIV care services [31]. A systematic review on family planning and HIV integration also found an 8% increase in contraceptive use [32]. Additionally, a 2020 South African study reported an increase in contraceptive prevalence among AGYW using PrEP from 62.3% at baseline to 74.5% during the trial [33]. These support the integration of SRH and HIV services, especially with community‐based, person‐centred service delivery models in addressing young women's reproductive health needs (Table 3).

Our study has several limitations. The duration of observation limits our ability to confirm long‐term trends in service utilization. We could not determine how many participants, particularly those diagnosed with HIV, had disengaged from care, which constrains our assessment of the true extent of service gaps. While our findings may not be generalizable to the entire country, the similar socio‐economic and healthcare contexts in rural settings across the region suggest potential applicability. Finally, although the peer navigator model seems to be an acceptable and feasible model of delivering PCC to AYA with a high unmet SRH and HIV care and prevention need, we need to await the final outcomes of the trial to understand its effectiveness and scalability. Despite these limitations, the study has notable strengths. The peer navigator‐led, community‐based approach enhanced access to SRH and HIV services among AYA by addressing disparities in healthcare access through equitable health needs assessments. Peer navigators provided person‐centred, personalized psychosocial support, which resulted in successful linkage to healthcare. Additionally, the comprehensive SRH and HIV serostatus neutral service delivery model simultaneously addressed multiple unmet health needs.

## 5 CONCLUSIONS

Community‐based and person‐centred approaches delivered through trained peer‐navigators are acceptable and feasible to reach AYA, including young men, with unmet SRH and HIV prevention and care needs and mobilize them into mobile SRH services. Providing HIV services with mobile SRH clinics creates demand for PrEP and supports ART initiation among AYA at high risk. These preliminary findings suggest that task shifting PCC to trained and supervised peer‐navigators could be a vital component of HIV prevention strategies, particularly for key populations and can fill unmet sexual health and social needs. After trial completion, we will evaluate the effectiveness of this intervention package in improving population‐level health outcomes and reducing sexually transmissible HIV among young people.

## COMPETING INTERESTS

The authors declare that they have no competing interests.

## AUTHORS’ CONTRIBUTIONS

MS conceived the study. MS, AC, KB, JB, NC, TZ, JD, CH, NO and JS designed the study. JB, AC, MS, JD and KB have access to the study data. JB conducted the analysis. JB and NN wrote the first draft of the manuscript. JB, NN, MS, KB, AC, TZ, LL, JS, TS and NC read and critically revised the manuscript. All authors read and approved the final manuscript.

## FUNDING

This trial was made possible through funding from the Bill and Melinda Gates Foundation (INV‐033650); the US National Institute of Health (NIH) R01 (5R01MH114560‐03); Africa Health Research Institute is supported by core funding from the Wellcome Trust (Core grant number082384/Z/07/Z). MS is an NIHR Research Professor (NIHR 301634). NC is supported by a Wellcome Trust Early Career Fellowship (grant number 224309/Z/21/Z). For the purpose of open access, the author has applied a CC BY public copyright licence to any Author Accepted Manuscript version arising from this submission. The funders have played no role in the study design, writing of the manuscript and in the decision to submit the manuscript for publication.

## Supporting information




**Table S1**: Prevalence of STIs by sex.
**Table S2**: Prevalence of STIs by sex and age group.

## Data Availability

Data sharing will be available upon completion of the study. All the protocols, study tools, data and data analysis plans from the study and the findings will be made available in compliance with the Bill and Melinda Gates Foundation's data sharing and open access policy. All data are collected, maintained, and analysed under the local IRB‐approved Policies and Procedures for data access and sharing, which will be made available in the AHRI repository at the time of publication of the primary outcome paper.
