# Supplementary material for: Person‐centred HIV care and prevention for youth in rural South Africa: preliminary implementation findings from Thetha Nami ngithethe nawe stepped‐wedge trial of peer‐navigator mobilization into mobile sexual health services
Source: J Int AIDS Soc. 2025 Oct 8;28(Suppl 5):e70032. doi: 10.1002/jia2.70032 (PMC12505191; doi:10.1002/jia2.70032)
Supplement: Supplementary file 1 — Table S1: Prevalence of STIs by sex. Table S2: Prevalence of STIs by sex and age group. [file JIA2-28-e70032-s001.docx]

**Thetha nami ngithethe nawe: 06 June 2022 – 18 September 2023**

**Supplementary Table 1 Prevalence of STIs by sex**

| **STIs** | **Female: Prevalence (95% CI)** | **Male: Prevalence (95% CI)** | **Overall: Prevalence (95% CI)** |
| --- | --- | --- | --- |
| N | 838 | 592 | 1430 |
| Any STIs | 37.2% (34.4%, 40.1%) | 17.9% (15.4%, 20.8%) | 29.2% (26.9%, 31.7%) |
| Chlamydia | 29.6% (26.4%, 33.0%) | 16.7% (14.3%, 19.5%) | 24.3% (22.1%, 26.6%) |
| Gonorrhoea | 9.2% ( 7.1%, 11.8%) | 3.4% ( 2.3%, 4.9%) | 6.8% ( 5.5%, 8.2%) |
| Trichomonas | 7.4% (5.1%, 10.6%) | 0%, n=8* | 7.3% ( 5.7%, 9.3%) |

***** Males were generally not tested for trichomonas in our study clinics

**Supplementary Table 2 Prevalence of STIs by sex and age group**

|  | **Age** | **Female** | | **Male** | | **Overall** | |
| --- | --- | --- | --- | --- | --- | --- | --- |
|  |  | **n** | **% (95% CI)** | **n** | **% (95% CI)** | **n** | **% (95% CI)** |
| **Any STIs** | 15-19 | 274 | 39.8% (35.3% - 44.4%) | 211 | 14.2% (10.1% - 19.6%) | 485 | 28.7% (24.4% - 33.3%) |
|  | 20-24 | 302 | 41.4% (37.2% - 45.7%) | 211 | 22.3% (16.0% - 30.1%) | 513 | 33.5% (31.2% - 35.9%) |
|  | 25-30 | 262 | 29.8% (23.9% – 36.4%) | 170 | 17.1% (12.7% - 22.5%) | 432 | 24.8% (20.8% - 29.2%) |
| **Chlamydia** | 15-19 | 274 | 34.7% (30.4% - 39.2%) | 211 | 14.2% (10.1% - 19.6%) | 485 | 25.8% (21.8% - 30.2%) |
|  | 20-24 | 302 | 33.1% (26.8% - 40.1%) | 211 | 20.9% (15.0% - 28.2%) | 513 | 28.1% (24.8% - 31.5%) |
|  | 25-30 | 262 | 20.2% (14.8% - 26.9%) | 170 | 14.7% (10.5% -20.2%) | 432 | 18.1% (14.6% - 22.1%) |
| **Gonorrhoea** | 15-19 | 274 | 10.6% (7.0% - 15.6%) | 211 | 2.4% (0.9% -6.2%) | 485 | 7.0% (4.8% - 10.1%) |
|  | 20-24 | 302 | 9.6% (7.2% - 12.8%) | 211 | 2.8% (1.3% - 6.2%) | 513 | 6.8% (5.2% - 8.9%) |
|  | 25-30 | 262 | 7.3% (3.9% - 13.0%) | 170 | 5.3% (2.7% - 10.2%) | 432 | 6.5% (4.2% - 9.9%) |
| **Trichomonas** | 15-19 | 274 | 6.6% (3.5% - 12.0%) | 4 | 0% | 278 | 6.5% (3.4% - 11.9%) |
|  | 20-24 | 302 | 8.3% (5.5% - 12.3%) | 3 | 0% | 305 | 8.2% (5.4% - 12.2%) |
|  | 25-30 | 261 | 7.3% (4.8% - 10.9%) | 1 | 0% | 262 | 7.3% (4.8% - 10.9%) |
